# The Decrement of Hemoglobin Concentration with Angiotensin II Receptor Blocker Treatment Is Correlated with the Reduction of Albuminuria in Non-Diabetic Hypertensive Patients: Post-Hoc Analysis of ESPECIAL Trial

**DOI:** 10.1371/journal.pone.0128632

**Published:** 2015-06-22

**Authors:** Jung Nam An, Jin Ho Hwang, Jung Pyo Lee, Ho Jun Chin, Sejoong Kim, Dong Ki Kim, Suhnggwon Kim, Jung Hwan Park, Sung Joon Shin, Sang Ho Lee, Bum Soon Choi, Chun Soo Lim

**Affiliations:** 1 Department of Internal Medicine, Seoul National University Boramae Medical Center, Seoul, Korea; 2 Department of Internal Medicine, Seoul National University College of Medicine, Seoul, Korea; 3 Department of Internal Medicine, Chung-Ang University Hospital, Seoul, Korea; 4 Department of Internal Medicine, Seoul National University Bundang Hospital, Seong-Nam, Korea; 5 Department of Internal Medicine, Konkuk University School of Medicine, Seoul, Korea; 6 Department of Internal Medicine, Dongguk University Ilsan Hospital, Goyang, Korea; 7 Department of Internal Medicine, Kyung Hee University Hospital at Gangdong, Seoul, Korea; 8 Department of Internal Medicine, Seoul St. Mary’s Hospital, Seoul, Korea; University of Perugia, ITALY

## Abstract

Blockade of the renin-angiotensin-aldosterone system exhibits a renoprotective effect; however, blockade of this system may also decrease hemoglobin (Hb) and erythropoietin (EPO) levels. We evaluated the correlation between reduced albuminuria and decreased hemoglobin concentrations after treatment with an angiotensin II receptor blocker (ARB). Two hundred forty-five non-diabetic hypertensive participants with established albuminuria and relatively preserved renal function were treated with an ARB (40 mg/day olmesartan) for eight weeks. Subsequent changes in various clinical parameters, including Hb, EPO, and albuminuria, were analyzed following treatment. After the 8-week treatment with an ARB, Hb and EPO levels significantly decreased. Patients with a greater decrease in Hb exhibited a greater reduction in 24-hour urinary albumin excretion compared with patients with less of a decrease or no decrease in Hb, whereas no associations with a decline in renal function and EPO levels were noted. Multivariate logistic regression analysis demonstrated a correlation between the reduction of urine albumin excretion and the decrease in Hb levels (after natural logarithm transformation, adjusted odds ratio 1.76, 95% confidence interval 1.21-2.56, *P* = 0.003). Linear regression analysis also supported this positive correlation (Pearson correlation analysis; R = 0.24, *P* < 0.001). Decreased Hb concentrations following ARB treatment were positively correlated with reduced albuminuria in non-diabetic hypertensive patients, regardless of decreased blood pressure and EPO levels or renal function decline.

## Introduction

Blockade of the renin-angiotensin-aldosterone system (RAAS) has a crucial role in preventing progressive renal dysfunction and cardiovascular morbidity and mortality by lowering blood pressure (BP) and reducing proteinuria [1–4]. Angiotensin II receptor blockers (ARBs) and angiotensin-converting enzyme inhibitors (ACEIs) are considered pivotal treatments for diabetic and non-diabetic patients with chronic kidney disease (CKD), largely due to their renoprotective and cardioprotective effects [5–7].

In addition to these beneficial effects, several adverse effects related to the use of ARBs or ACEIs have been reported, including dry cough, angioedema, and hyperkalemia. Another adverse effect involves decreased hemoglobin (Hb) levels. Several previous reports demonstrated that ACEIs and ARBs decrease Hb concentrations with a significant reduction in erythropoietin (EPO) levels in patients with normal renal function [8], on renal replacement therapy, and subject to kidney transplantation [9–13]. Danovitch *et al*. documented the dose-dependent effects of RAAS blockade on decreased EPO and Hb levels [14], and the combination of one or more RAAS blockade medications is also associated with a greater decrease in Hb concentrations as well as a greater antiproteinuric effect [15]. In addition, an experimental study reported that ARBs exhibit a more severe suppressive effect on erythropoiesis compared with ACEIs [16].

However, most of these studies were conducted in relatively small study populations with diabetes mellitus or overt kidney disease, furthermore, the correlation between the reduction in albuminuria and the decrement in hemoglobin level has not been assessed thoroughly.

In this study, we aimed to determine the lowering effects of an RAAS blockade medication (olmesartan) on Hb concentrations and investigate the main factors related to decreased hemoglobin levels, in particular, the correlation or cause-effect relationship between reduced Hb concentrations and albuminuria in non-diabetic hypertensive patients with albuminuria.

## Materials and Methods

### Study population

This is a post-hoc analysis of an open-label, case-control, randomized clinical trial (clinicaltrials.gov registration number NCT01552954, ESPECIAL trial). The study design and protocol, the inclusion and exclusion criteria, and the primary outcome are described in detail elsewhere [17]. In short, 245 non-diabetic hypertensive patients over the age of 19 years with a modification of diet in renal disease (MDRD) estimated glomerular filtration rate (eGFR) ≥ 30 ml/min/1.73 m^2^, a random urine albumin-to-creatinine ratio ≥ 30 mg/g, and creatinine levels measured more than twice with an interval of one week or more in the last 6 months from the outpatient renal clinics of 7 centers in Korea were enrolled between March 2012 and March 2013.

### Study protocol

After the run-in period (week 0), all patients were treated with 40 mg/day olmesartan medoxomil during the overall study period. At 8 weeks, eligible patients were randomly assigned to receive either intensive low salt diet (LSD) education or conventional LSD education during the subsequent 8 weeks (the intensive group received a weekly 30-minute dietary consultation and feedback by phone; the conventional group received typical education at the outpatient clinic). Physical and laboratory examinations were conducted at an 8-week interval. The primary outcome was the decrease in 24-hour urinary albumin excretion after LSD intervention. The study was conducted in accordance with the 2008 Declaration of Helsinki and the guidelines for good clinical practice. This study was approved by 7 Institutional Review Board: Seoul National University Hospital, Seoul National University Bundang Hospital, Seoul National University Boramae Medical Center, Konkuk University Hospital, Kyung Hee University Hospital at Gangdong, Dongguk University Ilsan Hospital, and Seoul St. Mary’s Hospital. All participants provided written informed consent before inclusion in the study.

### Data collection

Demographics and baseline clinical characteristics, including lifestyle, co-morbid diseases, and concurrent medications, were assessed at the initiation of olmesartan treatment. Hb; serum creatinine (sCr); eGFR; EPO; 24-hour urine excretion of albumin, sodium, and creatinine; and creatinine clearance (Ccr) were obtained at 0 and 8 weeks, evaluated, and analyzed. SCr levels were measured by an assay based on isotope dilution mass spectrometry (IDMS), and eGFR were calculated using the following IDMS-traceable Modification of Diet in Renal Disease equation: GFR (ml/min/1.73 m2) = 175 x (sCr)^-1.154^ x (age in years)^-0.203^ x (0.742 if female) [18].

### Statistical analysis

Categorical variables described as frequency and proportion, were compared using the chi-square test; continuous variables expressed as the mean ± standard deviation or median (interquartile range) were compared using Student's *t*-test or the Mann–Whitney *U* test according to the normality assumption. After the test of normality, 24-hour urine albumin excretion was transformed into natural logarithms, and then was analyzed. A simple logistic regression model was used to determine the unadjusted odds ratios (ORs) and 95% confidence intervals (CIs). A correlation analysis was conducted to avoid multi-collinearity; only one variable in highly correlated variable sets was selected for multiple logistic regression analysis. Statistically significant covariables from the univariate analysis and clinically important covariables were included in the final multiple logistic regression model, which was conducted in a backward stepwise manner. A *P*-value of less than 0.05 was considered statistically significant. Statistical analysis was performed with SPSS version 20.0 K software (SPSS Inc., Chicago, IL, USA).

## Results

### Baseline characteristics and laboratory findings

The mean age of the patients was approximately 50 years, and 122 patients (49.8%) were male. We compared the patients’ laboratory findings before and after the 8-week ARB treatment (Table 1). Systolic and diastolic BP decreased following ARB treatment, and Hb and EPO levels also significantly decreased during the eight weeks of the study. Hb levels decreased from 13.9 ± 1.7 g/dL to 13.6 ± 1.7 g/dL, and EPO levels decreased from 17.2 ± 12.1 U/L to 14.9 ± 14.4 U/L. In contrast, renal function, as measured by sCr and eGFR, was not affected by the administration of ARB. Among the results measured using urine samples, 24-hour urine sodium excretion and Ccr did not differ during the study period; however, the median amount of albumin excreted in urine over 24 hours decreased from 565.0 mg/day to 281.0 mg/day (*P* < 0.001) after treatment with ARB.

**Table 1:** Baseline characteristics and laboratory findings according to study period.

	Baseline (0^th^ week)	After 8 week	*P*
Age (years)	49.5 ± 13.3		
Male gender (n, %)	122 (49.8)		
Dyslipidemia (n, %)	135 (55.1)		
Body mass index (kg/m^2^)	25.4 ± 3.8		
Systolic BP (mmHg)	130.9 ± 11.8	122.6 ± 14.4	<0.001
Diastolic BP (mmHg)	79.4 ± 9.1	73.9 ± 10.3	<0.001
**Blood measurements**			
WBC (x /mm^3^)	6387.5 ± 1737.3	6428.8 ± 1672.5	0.610
Hemoglobin (g/dL)	13.9 ± 1.7	13.6 ± 1.7	0.049
Hematocrit (%)	40.9 ± 4.6	40.2 ± 4.6	0.158
Platelet (x1000/mm^3^)	239.8 ± 60.2	232.3 ± 53.4	0.229
BUN (mg/dL)	17.2 ± 6.3	19.2 ± 7.8	0.005
Creatinine (Cr) (mg/dL)	1.1 ± 0.4	1.2 ± 0.4	0.277
eGFR (mL/min/1.73m^2^)	67.3 ± 24.6	64.7 ± 24.4	0.242
Cholesterol (mg/dL)	183.8 ± 35.2	175.0 ± 31.7	0.010
Uric acid (mg/dL)	6.4 ± 1.8	6.8 ± 1.8	0.009
Na^+^ (mEq/L)	140.7 ± 2.2	140.5 ± 2.4	0.719
K^+^ (mEq/L)	4.3 ± 0.4	4.5 ± 0.4	<0.001
Cl^-^ (mEq/L)	104.1 ± 3.8	104.5 ± 2.9	0.323
TCO_2_ (mEq/L)	26.4 ± 2.8	25.7 ± 2.8	0.012
EPO (U/L)	17.2 ± 12.1	14.9 ± 14.4	<0.001
**Urine measurements**			
24-h urine Na^+^ (mEq/day)	155.2 ± 70.0	156.5 ± 72.7	0.998
24-h urine K^+^ (mEq/day)	54.4 ± 21.9	52.9 ± 19.7	0.604
24-h urine Cr (mg/day)	1226.9 ± 422.3	1211.3 ± 415.8	0.636
24-h urine albumin (mg/day)	565.0 (242.7–1285.3)	281.0 (104.2–640.3)	<0.001
Cr clearance (mL/min)	80.8 ± 34.1	77.0 ± 34.3	0.125

All data are expressed as mean ± standard deviation or median (interquartile range).

BP, blood pressure; BUN, blood urea nitrogen; EPO; erythropoietin; GFR, glomerular filtration rate

### Comparison according to decreased hemoglobin levels

**Fig 1:**
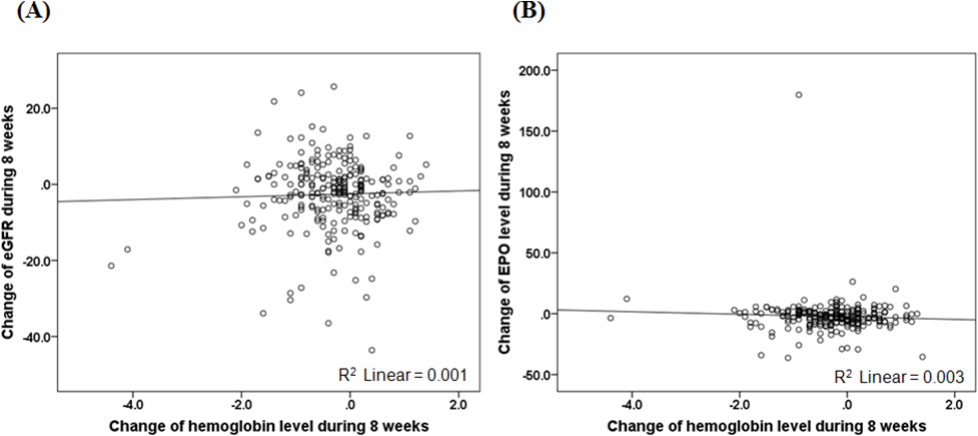
Correlation between the decrease in hemoglobin level and the decline in eGFR levels. **A.** Changes in eGFR levels following angiotensin receptor blocker treatment do not correlate with decreased hemoglobin levels (*P* = 0.627). GFR, glomerular filtration rate. **B**. Correlation between the decrease in hemoglobin level and the decline in EPO levels. Changes in EPO levels following angiotensin receptor blocker treatment do not correlate with decreased hemoglobin levels (*P* = 0.378). EPO, erythropoietin.

To examine various clinical parameters associated with changes in Hb levels, all patients were classified into two groups based on the mean decrease in Hb levels during the 8-week ARB treatment. In the group that exhibited a greater decrease in Hb levels, increased numbers of current smokers and individuals with a history of taking aspirin and statins were noted (Table 2). Parameters measured at week 0 were not related to the decrease in Hb levels, with the exception of serum cholesterol levels. Patients in the group with a greater decrease exhibited lower BP and EPO levels at week 8 and a greater reduction in systolic BP between weeks 0 and 8 compared with the group with less of a decrease (Table 3). In addition, a greater reduction in 24-hour urinary albumin excretion was significantly associated with a greater decrease in Hb levels. By contrast, no associations were noted among decreased Hb levels and the extent of decline in eGFR, Ccr, and EPO levels. These findings were also verified by linear regression analyses (Fig 1A and 1B).

**Table 2:** Baseline characteristics and laboratory findings according to the decrement of hemoglobin level.

	Lesser decrease or increase group (-1.4~0.2 g/dL) (N = 126)	Greater decrease group (0.3~4.4 g/dL) (N = 119)	*P*
Age (years)	50.2 ± 13.0	48.8 ± 13.7	0.351
Male gender (n, %)	64 (50.8)	58 (48.7)	0.748
Body mass index (kg/m^2^)	25.4 ± 4.3	25.1 ± 3.9	0.248
Current smoker (n, %)	11 (8.7)	18 (15.1)	0.026
Smoking amount (pack-years)	7.1 ± 13.4	4.1 ± 9.2	0.251
Dyslipidemia (n, %)	71 (58.2)	64 (56.6)	0.809
Treated with aspirin (n, %)	51 (40.5)	28 (23.5)	0.005
Treated with statin (n, %)	70 (55.6)	51 (42.9)	0.047
Systolic BP (mmHg)	131.7 ± 11.3	130.0 ± 12.2	0.122
Diastolic BP (mmHg)	79.5 ± 9.0	79.4 ± 9.3	0.712
Hemoglobin (g/dL)	13.7 ± 1.6	14.1 ± 1.8	0.058
Hematocrit (%)	40.3 ± 4.2	41.4 ± 4.8	0.077
Creatinine (Cr) (mg/dL)	1.1 ± 0.4	1.2 ± 0.5	0.902
eGFR (mL/min/1.73m^2^)	67.4 ± 23.5	67.1 ± 25.9	0.890
Cholesterol (mg/dL)	177.5 ± 31.7	190.5 ± 37.5	0.005
Uric acid (mg/dL)	6.4 ± 1.8	6.4 ± 1.9	0.900
Na^+^ (mEq/L)	140.7 ± 2.2	140.7 ± 2.2	0.989
K^+^ (mEq/L)	4.3 ± 0.4	4.3 ± 0.4	0.084
Cl^-^ (mEq/L)	103.9 ± 4.7	104.3 ± 2.7	0.576
TCO_2_ (mEq/L)	26.6 ± 2.7	26.2 ± 2.8	0.373
EPO (U/L)	17.9 ± 13.4	16.4 ± 10.6	0.222
24-h urine Na^+^ (mEq/day)	155.8 ± 66.7	154.4 ± 73.6	0.496
24-h urine K^+^ (mEq/day)	54.7 ± 21.8	54.0 ± 22.0	0.957
24-h urine Cr (mg/day)	1225.0 ± 407.5	1228.9 ± 439.1	0.740
24-h urine albumin (mg/day)	488.2 (210.0–1171.0)	715.2 (330.6–1366.0)	0.101
Cr clearance (mL/min)	81.9 ± 32.5	79.6 ± 35.9	0.447

All data are expressed as mean ± standard deviation or median (interquartile range).

BP, blood pressure; BUN, blood urea nitrogen; EPO; erythropoietin; GFR, glomerular filtration rate

**Table 3:** Laboratory findings at 8^th^ week according to the decrement of hemoglobin level.

	Lesser decrease or increase group (-1.4~0.2 g/dL) (N = 126)	Greater decrease group (0.3~4.4 g/dL) (N = 119)	*P*
Systolic BP (mmHg)	125.2 ± 14.6	119.7 ± 13.7	0.002
0^th^-8^th^ Systolic BP (mmHg)	6.5 ± 16.1	10.3 ± 14.3	0.027
Diastolic BP (mmHg)	75.8 ± 10.5	71.3 ± 11.7	0.001
Hemoglobin (g/dL)	13.9 ± 1.7	13.2 ± 1.7	0.002
0^th^-8^th^ Hemoglobin (g/dL)	-0.2 ± 0.4	0.9 ± 0.6	<0.001
Hematocrit (%)	41.0 ± 4.4	39.3 ± 4.7	0.004
BUN (mg/dL)	18.3 ± 6.1	20.3 ± 9.1	0.321
Creatinine (mg/dL)	1.2 ± 0.4	1.2 ± 0.5	0.991
eGFR (mL/min/1.73m^2^)	64.6 ± 23.0	64.8 ± 26.0	0.897
0^th^-8^th^ eGFR (mL/min/1.73m^2^)	2.9 ± 8.0	2.3 ± 10.4	0.491
EPO (U/L)	15.2 ± 9.5	14.6 ± 18.2	0.010
0^th^-8^th^ EPO (U/L)	2.7 ± 8.2	1.8 ± 18.5	0.831
24-h urine Na^+^ (mEq/day)	156.0 ± 70.3	157.0 ± 75.5	0.890
0^th^-8^th^ 24-h urine Na^+^(mEq/day)	-0.2 ± 70.9	-2.9 ± 66.5	0.769
24-h urine albumin (mg/day)	279.3 (104.2–737.0)	288.2 (101.0–597.0)	0.575
0^th^-8^th^ 24-h urine albumin (mg/day)	126.1 (21.0–454.4)	317.5 (110.0–933.0)	<0.001
Cr clearance (mL/min)	76.2 ± 34.1	77.8 ± 34.6	0.534
0^th^-8^th^ Cr clearance (mL/min)	5.3 ± 16.9	1.6 ± 20.1	0.091

All data are expressed as mean ± standard deviation or median (interquartile range).

BP, blood pressure; BUN, blood urea nitrogen; EPO; erythropoietin; GFR, glomerular filtration rate

### Comparison according to albuminuria reductions

Next, we divided all participants into 2 groups based on 50% reduction in 24-hour urine excretion of albumin to investigate the correlation of reduced albuminuria with other clinical parameters, particularly decreased Hb levels (Table 4). Patients in the group exhibiting a greater reduction in albuminuria were characterized with a younger age, lower systolic and diastolic BP, larger reduction in systolic BP, and a considerably greater decrease in 24-hour urine sodium excretion following 8-week ARB treatment compared with the other group. Moreover, lower Hb levels at week 8 and a greater decrease in Hb levels and renal function were significantly associated with a greater reduction in albuminuria. On the other hand, decreased EPO levels were not correlated with the change in urinary albumin excretion.

**Table 4:** Comparison according to the reduction in albuminuria for 8 weeks.

	Lesser reduction group (< 50%) (N = 129)			Greater reduction group (≥ 50%) (N = 114)			*P* ^*a*^	*P* ^*b*^	*P* ^*c*^
	0^th^ week	8^th^ week	0^th^– 8^th^	0^th^ week	8^th^ week	0^th^– 8^th^			
Age	51.7 ± 12.1			46.9 ± 14.4			0.005		
Systolic BP (mmHg)	131.3 ± 12.3	126.5 ± 14.0	4.8 ± 14.9	130.5 ± 11.3	118.3 ± 13.6	12.2 ± 15.0	0.845	<0.001	<0.001
Diastolic BP (mmHg)	80.3 ± 9.3	76.2 ± 10.7	4.1 ± 11.1	78.5 ± 8.8	71.5 ± 9.2	7.0 ± 10.3	0.069	<0.001	0.128
Hemoglobin (g/dL)	14.0 ± 1.8	13.8 ± 1.7	0.1 ± 0.7	13.8 ± 1.7	13.3 ± 1.7	0.5 ± 0.8	0.517	0.018	<0.001
Hematocrit (%)	41.0 ± 4.7	40.8 ± 4.7	0.2 ± 1.9	40.7 ± 4.5	39.5 ± 4.5	1.2 ± 2.2	0.513	0.013	<0.001
eGFR (mL/min/1.73m2)	68.1 ± 25.3	67.1 ± 25.5	1.0 ± 8.3	66.3 ± 23.8	61.9 ± 22.7	4.4 ± 9.9	0.602	0.133	0.002
EPO (U/L)	17.1 ± 13.3	15.2 ± 10.6	1.9 ± 7.5	17.2 ± 10.8	14.7 ± 17.8	2.5 ± 19.1	0.648	0.173	0.059
24-h urine Na^+^ (mEq/day)	154.1 ± 68.4	172.2 ± 77.0	-18.1 ± 66.5	155.4 ± 71.8	139.0 ± 64.0	16.4 ± 66.4	0.828	0.001	<0.001
24-h urine albumin (mg/day)	523.0 (158.0–1149.5)	490.0 (166.9–896.7)	62.8 (-16.8–234.0)	675.0 (316.0–1508.0)	197.2 (80.0–389.0)	454.2 (214.0–1041.0)	0.039	<0.001	<0.001
Cr clearance (mL/min)	80.8 ± 36.8	79.3 ± 34.8	0.9 ± 17.6	81.1 ± 31.1	74.4 ± 33.6	6.3 ± 19.2	0.701	0.275	0.001

All data are expressed as mean ± standard deviation or median (interquartile range).

^a^ *P*-value for comparison between lesser- and greater- reduction group at baseline

^b^ *P*-value for comparison between lesser- and greater- reduction group after 8 weeks

^c^ *P*-value for comparison of the changes during 8 weeks between lesser- and greater- reduction group

BP, blood pressure; EPO; erythropoietin; GFR, glomerular filtration rate

### Correlation between albuminuria reduction and decreased hemoglobin levels

**Fig 2:**
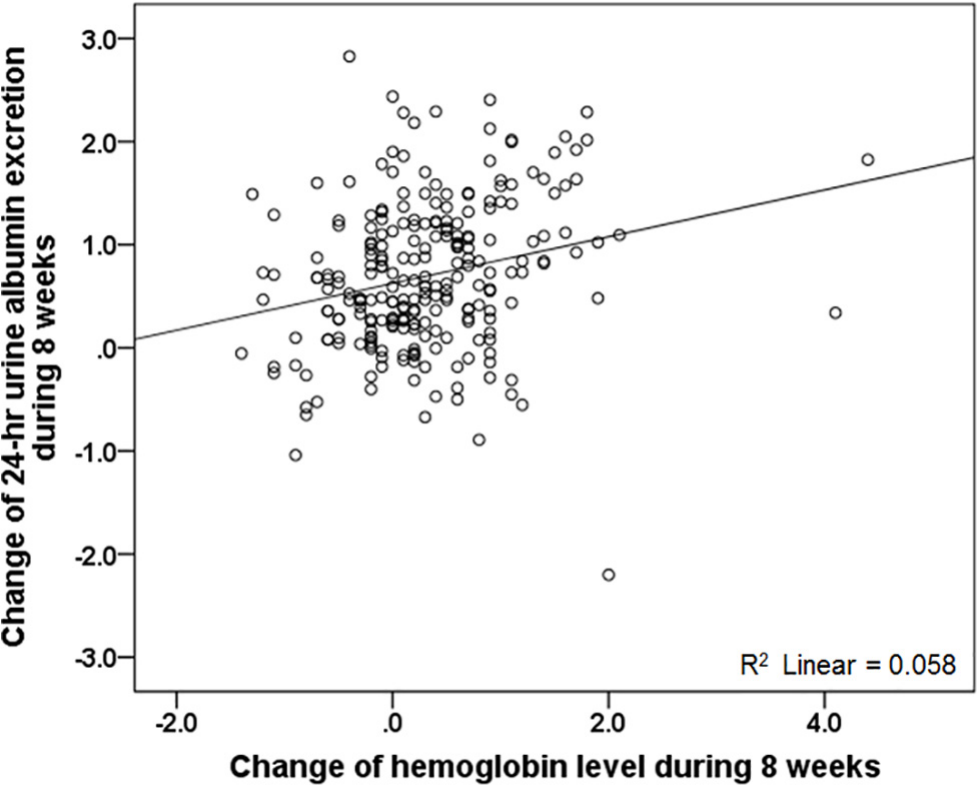
Correlation between the reduction in 24-hour urine albumin excretion and hemoglobin levels. Hemoglobin levels significantly decreased as the 24-hour urine albumin excretion decreased (Pearson’s correlation analysis; R = 0.24, *P* < 0.001).

To determine the effect of reduced urine albumin excretion on decreased Hb levels, we performed multivariate logistic regression analysis after natural logarithmic transformation of urinary albumin excretion (Table 5). As 24-hour urinary excretion of albumin decreased, the risk for decreased Hb levels increased, with an unadjusted OR of 1.71 (95% CI 1.18–2.48, *P* = 0.004). Model 3, which adjusted for age, gender, and the extent of the decrease in eGFR and systolic BP, demonstrated that the decrease in Hb levels was independently correlated with the reduction in albuminuria (adjusted OR 1.76, 95% CI 1.21–2.56, *P* = 0.003) (Table 6). Linear regression analyses also revealed a positive correlation between the two parameters (Pearson correlation analysis; R = 0.24, *P* < 0.001) (Fig 2).

**Table 5:** Multivariate logistic analysis for the decrement of hemoglobin level.

	Univariate analysis		Multivariate analysis	
	OR (95% CI)	*P*	OR (95% CI)	*P*
**Age (10-yr increment)**	0.92 (0.77–1.12)	0.413	0.97 (0.80–1.18)	0.785
**Male gender**	0.92 (0.56–1.52)	0.748	0.90 (0.54–1.51)	0.699
**0** ^**th**^ **-8** ^**th**^ **eGFR (per 10 mL/min/1.73m2)**	0.94 (0.72–1.24)	0.664	0.85 (0.64–1.14)	0.280
**0** ^**th**^ **-8** ^**th**^ **Systolic BP (per 10 mmHg)**	1.17 (0.99–1.39)	0.059	1.10 (0.92–1.31)	0.283
**0** ^**th**^ **-8** ^**th**^ **Ln (24-h urine albumin)**	1.71 (1.18–2.48)	0.004	1.76 (1.21–2.56)	0.003

BP, blood pressure; GFR, glomerular filtration rate

**Table 6:** Correlation between decrement of hemoglobin level and the reduction in albuminuria.

0^th^-8^th^ Ln (24-h urine albumin)	Multivariate logistic analysis		
	Model 1^*^	Model 2^†^	Model 3^‡^
**OR (95% CI)**	1.71 (1.18–2.48)	1.71 (1.18–2.48)	1.76 (1.21–2.56)
***P*-value**	0.004	0.004	0.003

^*^ Unadjusted model

^†^ Model 1 + adjustment for age, gender, and the difference in eGFR during 8 weeks

^‡^ Model 2 + adjustment for the difference in systolic blood pressure during 8 weeks

## Discussion

This study identified the relationship between treatment with an angiotensin II receptor blocker and decreased Hb levels. We also determined a correlation between decreased hemoglobin levels and reduced urine albumin excretion in non-diabetic hypertensive patients.

Several previous studies reported no association among ARBs with Hb and EPO levels [6,11,19–21], whereas 8-week ARB treatment significantly decreased Hb and EPO levels in this study. This finding has been well documented by prior studies [12,22–24].

The mechanisms involved in Hb level reductions related to ARBs are well known; erythropoiesis is inhibited as a result of a decrease in the peptide hormone angiotensin II (Ang II), which stimulates EPO secretion and acts as a growth factor for erythropoiesis in bone marrow [10,23,25–27]. These findings are also attributed to the reduced production of hypoxia inducible factor-1α (HIF-1α), which functions as a transcriptional factor for EPO production, as a result of increased renal blood flow following Ang II blockade [28–30]. Reportedly, Ang II directly exhibits a stimulating effect on sodium reabsorption in the proximal tubule. This effect induces enhanced tubulointerstitial O_2_ demand [28] as well as a selective vasoconstrictive effect on the efferent arterioles, which decreases O_2_ delivery to the tubule-interstitial compartment [29,31]. In several clinical and experimental studies, renal medullar hypoxia arising from the hemodynamic effects of Ang II is attenuated by the administration of Ang II receptor blockade medications [32–35]. In addition, decreased levels of insulin-like growth factor, which stimulates erythropoiesis [11,36], and inhibition of N-acetyl-seryl-aspartyl-proline catabolism, which decreases the proliferation of red cell precursors [37], are known actions of ARBs related to decreased Hb levels.

Above all, however, this study was characterized by a different level of significance compared with other studies [24,38,39] given that we demonstrated a positive correlation between the decrease in Hb level and the reduction in albuminuria regarding ARB treatment (especially, 40 mg/day olmesartan medoxomil), regardless of EPO level, BP, and eGFR. Our findings are also consistent with those of Inoue A *et al*., indicating that the decrease in Hb was not likely attributed to a deterioration in renal function following ARB treatment [40]. We proved this finding using multivariate logistic regression and linear regression analyses.

The reasons for this finding are unclear but may be related to the following facts. First, HIF-1α upregulation via Ang II stimulation [41–44] increases the expression of vascular endothelial growth factor (VEGF) in podocytes [45–47]. VEGF is a vital protein required for normal glomerular filtration barrier function [48–51] and for the development of proteinuria through vascular permeability modifications [47,52]. VEGF levels are markedly increased and correlated with the severity of proteinuria in diabetic nephropathy [53–56] and several types of glomerulonephritis [57–60]. Consequently, the inhibition of HIF-1α expression by an Ang II receptor blocker attenuates not only EPO-mediated erythropoiesis but also VEGF-mediated glomerular damage. Second, reactive oxygen species (ROS) induce endothelial cell dysfunction, subsequently leading to glomerular diseases, including diabetic nephropathy [61,62], as well as hypoxic conditions involved in EPO-mediated erythropoiesis. Ang II receptor blockers suppress these functions through decreased ROS production [29]. Collectively, the inhibition of Ang II-induced ROS production and HIF-1α-mediated VEGF expression might interactively contribute to decreased Hb levels and urine albumin excretion after ARB treatment, independent of the decrease of BP, EPO, and eGFR.

This finding has significant clinical implications. First, improvement of hypoxia induced by ARBs is one of the most important mechanisms associated with decreased albuminuria and prevention of CKD progression. In other words, the decrease in Hb levels appears not to be the adverse effect of ARBs but the beneficial effect accompanied by the renoprotective effect of ARBs. Second, EPO levels were lower in patients with a more significant decrease in Hb level; however, no quantitative correlation was noted between the reduction in EPO levels and decreased Hb, albuminuria, or renal function. These results indicate that decreased Hb levels are more closely related to reduced albuminuria rather than exclusively with decreased EPO levels. Third, the interrelation of decreased Hb and reduced albuminuria after adjustment for the impacts of BP or renal function on Hb levels through multivariate analysis imply that this correlation is not just because of the extent of RAAS blockade. Accordingly, our study suggests that the decrease in Hb levels may serve as a useful surrogate marker for the therapeutic effects of ARBs without the direct measurement of urine albumin excretion. Meanwhile, caution is required when monitoring hemoglobin levels in patients with a higher decrease in albuminuria following treatment with ARBs over approximately 8 weeks, regardless of renal function decline.

A few limitations to our study should be noted. First, this study did not include the control group who did not take ARB, therefore we cannot draw the conclusion that the correlation was caused solely by the use of ARB. Second, the comorbidities of the participants in this study were quite small in number and modest in severity. The number of concurrent medications, with the exception of anti-hypertensive drugs, was also minimal, and few medications affecting Hb concentrations were noted. For this reason, further investigations are needed to verify whether our findings are applicable to other patients or clinical situations with more severe comorbidities and the administration of significantly more medications compared with our study. Third, the original study was a randomized, controlled clinical trial, and we adjusted other factors through multivariate logistic regression analysis. However, confounding factors may be present in our results. Fourth, underlying renal diseases such as hypertension or chronic glomerulonephritis of study populations were not specified. Lastly, the long-term effects of these findings were not analyzed in this study. Nevertheless, when we analyzed the overall changes of parameters between weeks 0 and 16, similar results were noted and the influences of ARBs on albuminuria or Hb occur from 3 to 4 weeks after the initiation of ARB therapy. Thus, the changes for the first 8 weeks were sufficient to analyze and elucidate our findings.

Although additional research regarding the concrete and detailed mechanisms for our findings is needed, it is noteworthy that this is the first study to demonstrate a positive correlation between reductions in urine albumin excretion and Hb levels after ARB treatment.

In conclusion, the administration of angiotensin II receptor blocker therapy for 8 weeks significantly decreased Hb and EPO levels. The greater decrease in Hb levels was closely correlated with a greater reduction in albuminuria, regardless of the decrease of BP or the decline in renal function or EPO levels. Our findings suggest prominent preventative mechanisms for the progression of CKD caused by ARBs and the crucial clinical implications of ARB treatment in non-diabetic hypertensive patients.
